# Mining-Guided Machine Learning Analyses Revealed the Latest Trends in Neuro-Oncology

**DOI:** 10.3390/cancers11020178

**Published:** 2019-02-03

**Authors:** Taijun Hana, Shota Tanaka, Takahide Nejo, Satoshi Takahashi, Yosuke Kitagawa, Tsukasa Koike, Masashi Nomura, Shunsaku Takayanagi, Nobuhito Saito

**Affiliations:** Department of Neurosurgery, Graduate School of Medicine, The University of Tokyo, 7-3-1 Hongo, Bunkyo-ku, Tokyo 113-8655, Japan; thana-tky@umin.ac.jp (T.H.); tnejo-tky@umin.ac.jp (T.N.); takahashi-satoshi0719@g.ecc.u-tokyo.ac.jp (S.T.); yokitagawa-tky@umin.ac.jp (Y.K.); tkoike-ham@umin.ac.jp (T.K.); nomura-m@umin.ac.jp (M.N.); takayanagi-nsu@umin.ac.jp (S.T.); nsaito-tky@umin.net (N.S.)

**Keywords:** impact factor, machine learning, neuro-oncology, regression analysis, trend prediction, text-mining

## Abstract

In conducting medical research, a system which can objectively predict the future trends of the given research field is awaited. This study aims to establish a novel and versatile algorithm that predicts the latest trends in neuro-oncology. Seventy-nine neuro-oncological research fields were selected with computational sorting methods such as text-mining analyses. Thirty journals that represent the recent trends in neuro-oncology were also selected. As a novel concept, the annual impact (AI) of each year was calculated for each journal and field (number of articles published in the journal × impact factor of the journal). The AI index (AII) for the year was defined as the sum of the AIs of the 30 journals. The AII trends of the 79 fields from 2008 to 2017 were subjected to machine learning predicting analyses. The accuracy of the predictions was validated using actual past data. With this algorithm, the latest trends in neuro-oncology were predicted. As a result, the linear prediction model achieved relatively good accuracy. The predicted hottest fields in recent neuro-oncology included some interesting emerging fields such as microenvironment and anti-mitosis. This algorithm may be an effective and versatile tool for prediction of future trends in a particular medical field.

## 1. Introduction

In science, new research fields and methods are continuously evolving. The design and conduct of cutting-edge research mandate a keen perception of the current trends and future directions of the field. However, proper assessment of a large number of related literatures and information in a timely fashion would not necessarily be easy. A systematic review would be one solution; it prioritizes an overwhelmingly large number of research studies by their level of importance to retrieve the key information in a specific research field [1,2]. However, it will never be free from bias, given the nature of a study which involves manual data gathering. For that reason, a systematic review will not always be used for the decision-making process of researchers. Computer data mining is superior in coverage and objectivity for trend analysis in a specific research field. Attempts have already been made in various medical fields to predict future trends by machine learning analyses. In such studies, regression formulas are created from the obtained data to do so, but the prediction accuracy is yet to be satisfactory [3,4,5].

The field of neuro-oncology has undergone rapid progress in recent years. Treatment with tumor treating (TT) [6], glioma classification based on the tumor’s genomic abnormalities [7], and epigenetic dysregulation during the process of tumor initiation and progression [8] are some of the emerging fields. For any physician and researcher, appreciation of ‘hot topics’ in neuro-oncology is key to successful, future collaborative work.

**Figure 1:**
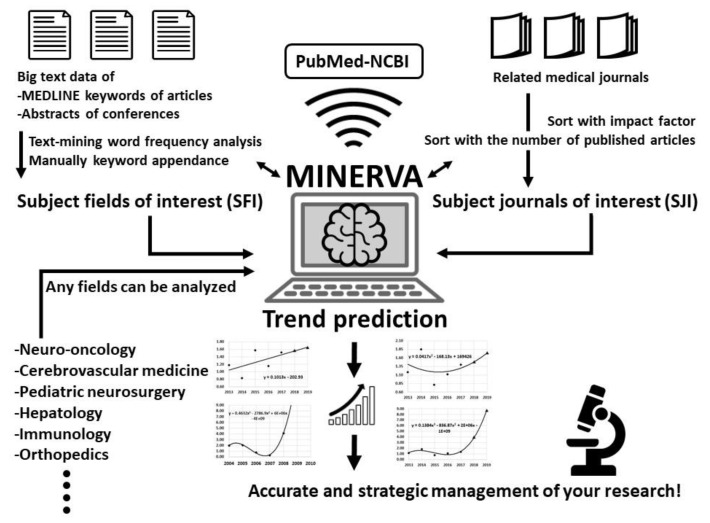
Conceptual diagram of Mining Integrated Navigation and Estimation Research Via Articles (MINERVA) algorithm. Based on the input data of subject fields of interest (SFI) and subject journals of interest (SJI), MINERVA will analyze the trends using PubMed database. By switching the input keywords, MINERVA can analyze the trends of any medical field.

We developed a novel algorithm, named Mining Integrated Navigation and Estimation Research Via Articles (MINERVA), which makes use of text mining and machine learning of a large amount of data extracted from the literature (Figure 1). In this study, the MINERVA algorithm was used to predict 20 neuro-oncology fields that are expected to make great leaps in 2019.

## 2. Results

### 2.1. Data Collection for Analysis

#### 2.1.1. Subject Fields of Interest

To analyze the trends of several research fields, the fields to be analyzed were first determined. Using the PubMed database (https://www.ncbi.nlm.nih.gov/pubmed/), all brain tumor-related articles published in 2017 were identified. Text-mining word frequency analysis was performed on text data of these articles and abstracts of neuro-oncological conferences. As a result, 79 keywords that seemed to represent recent trends in neuro-oncology were extracted as subject fields of interest (SFI) (Table 1).

#### 2.1.2. Subject Journals of Interest

Thirty subject journals of interest (SJI) (Table 1) that can be representative of trends in neuro-oncology were determined. They included 15 highest impact factor (IF) journals among 1935 journals that published at least one neuro-oncological article in 2017 and 15 journals with the largest number of published articles in 2017. Impact factors were obtained from the 2017 Journal Citation Reports [9].

#### 2.1.3. Annual Impact and Annual Impact Index

A system to objectively and sequentially evaluate the frequency and importance of SFI within the SJI is necessary. Therefore, the novel concepts of annual impact (AI) and annual impact index (AII) were employed in the current study. The AI of a certain subject field in a certain subject journal of a certain year was calculated according to the following formula:

AI=N×IF,

where N is the number of articles related to the subject field published in the subject journal in the year. The AII value of a certain subject field of a certain year was calculated according to the following formula:

AII=AI1+AI2+AI3+⋯+AI28+AI29+AI30,

where AI1 to AI30 are the AIs of each subject journal in that year. There were 30 journals in SJI, so numbers from 1 to 30 were assigned. In other words, the AII value of a certain subject field of a certain year is the sum of the AIs of each of the 30 journals for the year. Data of the number of articles that matched each condition were collected using PubMed. For example, the number of articles on “pilocytic astrocytoma” published in *Nature* in 2015 was obtained by entering the following command in the PubMed search window, and the number was 6.

((pilocytic astrocytoma) AND “Nature” [Journal]) AND (“2015” [Date-Publication]: “2015” [Date-Publication]).

Since the IF of *Nature* is 40.137, the AI of “pilocytic astrocytoma” in *Nature* in 2015 was calculated as follows.

AI=N×IF=6×40.137=240.822

Besides *Nature*, 25 journals contained articles on “pilocytic astrocytoma” in 2015. For example, there were two articles in the *Journal of the American Medical Association* (AI = 2 × 44.405 = 88.81) and one in *Science* (AI = 1 × 37.205 = 37.205). Thus, the AII of “pilocytic astrocytoma” in 2015 was 4068.629, as calculated by aggregating the 25 AIs according to the above mathematical rule.

### 2.2. Trend Predictions of Neuro-Oncology

#### 2.2.1. Mathematical Analyses

A total of 79 SFI fields were analyzed using a comparison of the rate of change of AII (Δ-AII). In other words, Δ-AII is one year’s AII change rate compared with the previous year. The Δ-AIIs of all 79 SFI fields were tracked for 10 years from 2008 to 2017. Three types of mathematical regression analyses based on the least-square method (linear prediction, quadratic polynomial, and cubic polynomial) were performed to predict trends in neuro-oncology in 2019. Regarding the prediction of the top 20 subject fields, we tried to predict the rankings for 1–3 years after collection of the data and also examined the accuracy rate of each.

#### 2.2.2. Accuracy of the Predictions by Multiple Regression Analyses

**Figure 2:**
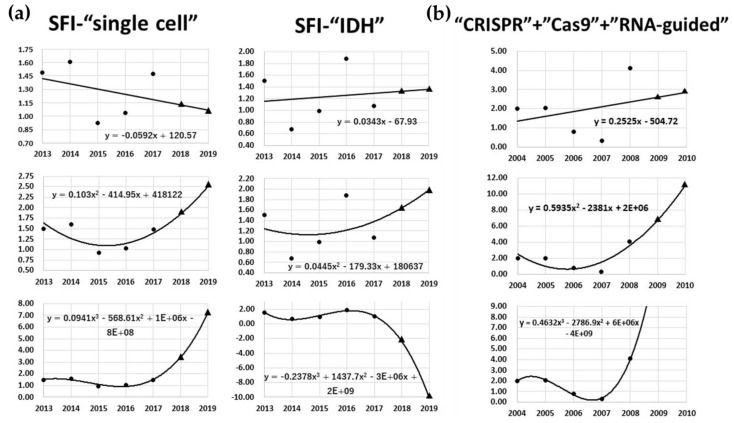
(**a**) Regression analytic graphs are exemplified. Left side shows the result of “single cell”, and the right shows “IDH”. The vertical axis is the fold value of Δ-AII. The upper row is the result of linear prediction, the middle is quadratic polynomial and the lower is cubic polynomial. The circles are actual measured values and the triangles are predicted values. (**b**) Regression analytic predictions of “CRISPR”, “Cas 9” and “RNA-guided”. The vertical axis is the fold value of Δ-AII. The analyzed AII is the sum of these three keywords’ AII. Other arrangements are the same as Figure 2a.

**Figure 3:**
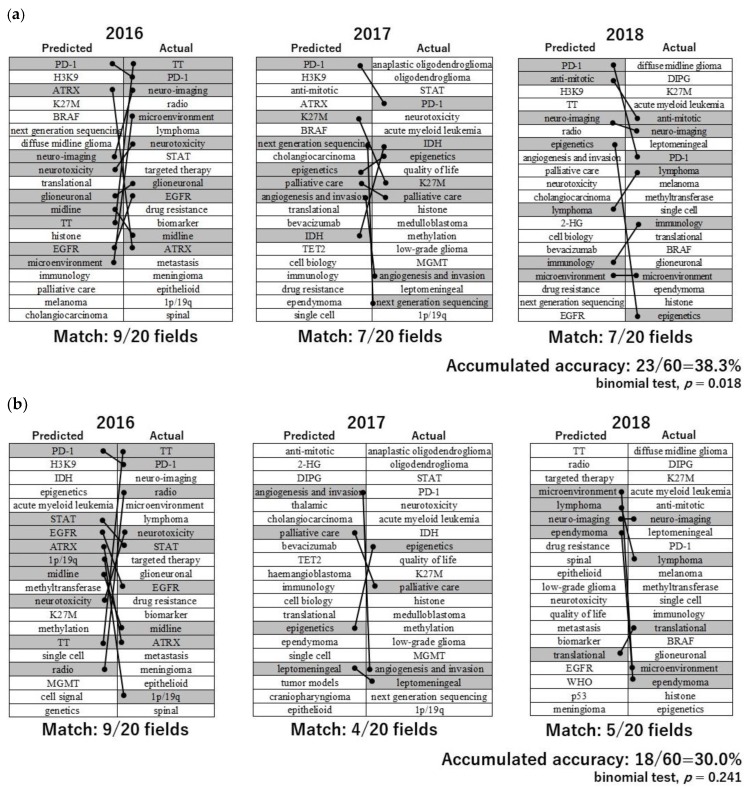
(**a**) The details of prediction with linear prediction using 5 years. The AII dataset to predict the top 20 fields with high Δ-AII of 2 years after the data collection period. Predicted top 20 fields with high Δ-AII are enumerated in left columns in order of expected Δ-AII scores. Right columns are the year’s actual top 20 fields with high Δ-AII in order of actual Δ-AII scores. Fields matching the prediction are connected by lines. The accumulated accuracy means Accuracy C of linear prediction of Table 2. (**b**) The details of prediction with quadratic polynomial. Other analytical conditions are the same as Figure 3a. Fields matching the prediction are connected by lines. The accumulated accuracy means accuracy C of quadratic polynomial of Table 2.

Regression analytic graphs are illustrated in Figure 2a, with “single cell” and “IDH” as examples. Predictions did not necessarily match between linear prediction, quadratic polynomial and cubic polynomial methods. Relatively accurate prediction was possible with the linear prediction method. With an accuracy of 70.6%, we were able to predict the Δ-AII of the following year of the data collection range within an error of 1.0-fold (Table 2). This means that the latest trends of about 56 out of 79 fields can be predicted with high accuracy. In addition, we were able to predict the top 20 fields with a high Δ-AII of 2 years after the data collection period with an accuracy of 38.3% (binomial test, *p* = 0.018), using linear prediction. Figure 3a shows the details of prediction. This method can constantly achieve a high-match rate of predictions. Interestingly, this method predicted a same field as highest expected field every year, “PD-1”. Although “PD-1” had never actually been ranked as highest, it had ranked in top 20 fields every year. “Cholangiocarcinoma”, which seems to have little relation to brain tumor, had been ranked sometimes. However, cholangiocarcinoma is known to have isocitrate dehydrogenase (IDH) mutation, and it had been frequently discussed with gliomas. On the other hand, the accuracy of quadratic polynomial with the same condition was 30.0% (binomial test, *p* = 0.241) (Figure 3b). This method achieved a high match rate in particular years, but as a whole was not such an accurate method. The results obtained with other analytical methods are shown in Table 2. Regarding the accuracy of B, C, and D, given that the coincidence match rate was 25.3% (20/79 = 0.253), most analytical methods tended to have higher predictive accuracy than by sheer coincidence, and some methods had statistical significance. Accuracy D of linear prediction was not statistically significant, but the *p*-value was 0.06. As a result of comprehensive judgment of these accuracy data, we concluded that linear prediction can be the most reliable prediction method.

#### 2.2.3. The Top 20 Hottest Fields of Neuro-Oncology in 2019

Based on the policy above, the top 20 hottest fields of neuro-oncology in 2019 were predicted using the linear prediction method and using the data of Δ-AII for the period of 2013–2017 (Table 3). The predicted rank was ordered by predicted Δ-AII scores. In the predicted fields, there are many emerging fields such as “microenvironment” and “anti-mitotic”, as well as some important fields already widely recognized, such as “immunology” and “epigenetics”. “Epigenetics”, “microenvironment”, “neurotoxicity”, “palliative care”, “anti-mitotic”, “angiogenesis and invasion”, “EGFR”, “lymphoma”, “TT” and “translational” had been repeatedly ranked in the top 20 fields in 2016–2018 with this method, in both predicted and actual fields (Figure 3a). Those four fields at the beginning, “epigenetics”, “microenvironment”, “neurotoxicity”, and “palliative care”, appeared a total of four times, which was the most frequent. This may reinforce the hopeful prospectivity of those fields in another form different from the predicted rank of 2019.

#### 2.2.4. Prediction of Milestone Discoveries in Other Fields

The current study focused on neuro-oncology, but the MINERVA algorithm can potentially be used to predict trends in any field by changing the input keywords. Meanwhile, the discovery of the CRISPR-Cas9 gene editing system in 2012 was a recent milestone in the field of medical biology [10]. We retrospectively assessed the ability of the MINERVA algorithm to predict its discovery in advance with the use of only three keywords for SFI: “CRISPR” “Cas 9” and “RNA-guided”. Then the following 15 journals dealing with cell engineering and genetics, or general science with the highest IFs were selected for SJI: *Nature Reviews*—*Molecular Cell Biology*, *Nature Biotechnology*, *Nature Reviews*—*Genetics*, *Nature*, *Science*, *Cell*, *Nature Genetics*, *Nature Reviews*—*Microbiology*, *Cell*—*Stem Cell*, *Nature Cell Biology*, *Cell Research*, *Trends in Cell Biology*, *Cell Host & Microbe*, *Annual Review of Cell and Developmental Biology*, and *Molecular Cell*. The AII data since 2000 were collected. As a result, a marked increase in the sum of the three keywords’ Δ-AII was predicted in 2008 by all three analytic models (Figure 2b). This means the possibility that an important discovery in the fields related to “CRISPR”, “Cas 9” and “RNA-guided” after 2009 could have been predicted as early as 2008. Individual keywords “CRISPR”, “Cas 9” and “RNA-guided” had been broadly used in the fields of archaea and genetics. However, by analyzing these three keywords together, it can be related to the trend of the CRISPR-Cas9 gene editing system. This prediction result indicates the good versatility of the MINERVA algorithm.

## 3. Discussion

In clinical and basic research, it is important to grasp the current status of the research field to predict future trends. This type of evaluation is often performed by the individual’s impression based on notable journals, conferences, and lectures. Scientific reporters have many similar personal impressions and tend to have subjective bias in their evaluations. The current study suggested that the MINERVA algorithm can be used to grasp current trends and predict at least some future trends in a specific research field. Notably, the MINERVA algorithm is characterized by its wide applicability; it can analyze any medical field simply by changing the selected keywords of SFI and SJI, whether it is another central nervous system disorder such as cerebrovascular disease or basic science, exemplified by our results regarding the CRISPR-Cas9 gene editing system.

Among the 20 fields predicted by the MINERVA algorithm, there were several fields of particular interest. Regarding the “anti-mitotic” field, the AII value was 25.353-fold greater than the value of the previous year in 2014. Likewise, the AII value of “TT” in 2015 also increased by 5.412-fold, so it would be reasonable to understand that the “anti-mitotic” field was paid attention to as the mechanism of the TT field [6]. Actually, an article about NovoTTF (Novocure, St. Heliar, Jersey) was responsible for the rapid increase in the “anti-mitotic” field in 2014 [11]. “Immunology” and “microenvironment” were ranked. Recently, many high-IF articles related to microenvironment and glioma have been published [12,13,14,15]. Tumor microenvironment is a concept related to micro cellular signals, receptors, structures, angiogenesis, molecules, etc. In particular, immunosurveillance systems are deeply related to tumor microenvironment, and many articles have recently been published which focus on immunology and microenvironment [13,15,16]. Considering that the word “microenvironment” appeared 146 times in the official abstracts of the Annual Meeting of the Society for Neuro-Oncology (SNO-2017, San Francisco, CA, USA) [17], we can see how this field has recently gained attention of neuro-oncologists. With respect to the field “epigenetics,” the discovery of mutation to the IDH1 and IDH2 genes in gliomas [18] has determined the prosperity of subsequent glioma and epigenetic studies. Even now, after 10 years have passed, important articles on gliomas and methylation as well as other epigenetic systems continue to be published [19,20,21]. It strongly suggests the importance of the field. The field “lymphoma” was a relatively unexpected result because there were relatively few titles in SNO-2017 pertaining to this subject. However, in 2015, seven articles including research on new drugs were published in high-IF journals [22,23,24,25,26,27,28], which seems to have contributed to its popularity.

As regards limitations to the current study, even though multiple regression analyses were performed, the prediction accuracy was not perfect. The prediction results of linear prediction, quadratic polynomial, and cubic polynomial are not always concordant (Figure 2a,b). In order to improve accuracy, advanced machine learning by artificial intelligence, such as deep learning, should be considered in future studies. In keyword selection, SFI and SJI are selected as objectively and extensively as possible, but it would be desirable to conduct analyses with a much larger number of keywords in SFI and SJI. In addition, compiling similar words in the current study is a work involving human intervention, which may have contained some degree of subjectivity. Natural language processing may achieve more objectivity in this process.

## 4. Material and Methods

### 4.1. Choosing the Subject Fields of Interest

With all brain tumor-related articles published in 2017, the MEDLINE information of all the articles was extracted as text data. Only Other Terms (OTs), but not medical subject headings (MeSHs), were subjected to word frequency analysis, because MsSHs are basically broad terms, so it was difficult to choose keywords that only appear in a specific field of brain tumors, whereas OTs include many keywords specific to the field of neuro-oncology, such as signal transducer and activator of transcription (STAT), IDH, and TT. For the extracted OT text data, text-mining word frequency analysis was performed using a publicly available program (https://textmining.userlocal.jp/) to select frequently used keywords. Next, 28 abstract categories of SNO-2017 were registered as 28 keywords [17]. Word frequency analysis of the English presentation titles of the SNO-2017 and the English symposium presentation titles of the Annual Meeting of the Japan Society of Brain Tumor Pathology (JSBTP-2017, Utsunomiya, Tochigi, Japan) was also performed and frequently cited keywords were extracted. In addition to these objectively extracted keywords, some keywords of brain tumor names were added manually to investigate the trends. Many synonyms and similar words that appeared as keywords in each data source were checked manually and then integrated into one keyword. We also manually excluded relatively vague keywords (e.g., survival, malignancy, etc.) that were judged as not suitable for tracking a specific research field of neuro-oncology. According to this method, 79 keywords that seemed to represent recent trends in neuro-oncology were extracted as SFI.

### 4.2. Data Collection with PubMed

Python 3.7.0 (https://www.python.org) was used to collect a large amount of journal data with PubMed. In addition, regarding the searching keywords, such as “genetics”, “single cell”, etc., which may lead to the identification of many other articles not related to neuro-oncology, the search was conducted by adding the term “brain tumor” as a keyword. By doing so, the directionality of the keywords was increased. About the possibility of retrieving the same article with multiple keywords, even if one article was found to be a related article of more than one keyword, we considered that it was not particularly problematic and did not affect the trend analysis of each subject field.

### 4.3. Mathematical Background of Δ-AII

The rule that Δ-AII is used for the analysis was adopted because inequality arises simply by comparing the AII values themselves. Each keyword has a difference in the “breadth” the word covers, and the “advantage” caused by the research scale to date. Such inequities need to be corrected. For example, the AII value of “epigenetics” in 2012 and 2013 were 15.760 and 88.141, respectively, and the Δ-AII for the period of 2012–2013 was calculated as 88.141/15.760 = 5.593. On the other hand, the Δ-AII of 2012–2013 of “glioblastoma” was similarly calculated as 3972.712/3298.539 = 1.204. Although the AII value was much higher for “glioblastoma”, “epigenetics” was judged as having more momentum than “glioblastoma” over this two-year period. In addition, when the AII value of a certain subject field is zero, it is not possible to calculate the rate of change. In such a case, 1.081 was substituted for zero. This number is the same score of one article of SJI’s lowest IF journal, “Child’s nervous system”.

### 4.4. Accuracy Validation of the Predictions

We validated the accuracy of the predictions by the three regression analyses using actual past data. Specifically, the Δ-AII data over a period of 5 years (year 1–5) were analyzed to predict the Δ-AII of the following year (year 6), which was then compared with the actual Δ-AII of year 6. We also rearranged the SFI fields in order of the predicted Δ-AII to determine the top 20 subject fields for each year. Similarly, the accuracy rate was calculated using the actual past data. Based on these analyses, we determined which regression method was most reliable for prediction.

### 4.5. Statistical Analyses

All statistical analyses were performed using JMP Pro 13 software (SAS Institute Inc., Cary, NC, USA) and R 3.4.1 [29]. The level of significance was set at a *p-*value of 0.05.

## 5. Conclusions

The current study highlights some evolving fields in clinical practice as well as research in neuro-oncology. In the upcoming few years, molecular biological approaches will become far more popular in neuro-oncological research. The fields of tumor microenvironment and epigenetics, both of which are closely related to many research fields like tumor immunology, will be more extensively discussed in the context of pathological mechanisms. In clinical practice, fine-grained treatments that emphasize the patient’s quality of life would be desirable, reflecting the current social situations. New therapeutic drugs, devices and techniques are constantly appearing. Clinicians and researchers are encouraged to remain keen on grasping the latest trends.

The MINERVA algorithm may provide useful data of current and future trends in the field of neuro-oncology. Moreover, it could potentially be applied to any medical research field by changing keywords. However, the accuracy of future prediction has room for improvement. A larger-scale system that takes into consideration more complex factors, such as deep learning by artificial intelligence, might be one future direction for better prediction.

## Figures and Tables

**Table 1:** Seventy-nine keywords of SFI and 30 journals of SJI.

SFI		SJI
1p/19q	lymphoma	*Cancer cell*
2-HG	medulloblastoma	*Cancer research*
acute myeloid leukemia	melanoma	*Cell*
anaplastic astrocytoma	meningioma	*Cell stem cell*
anaplastic oligodendroglioma	metabolism	*Child* *’s nervous system*
angiogenesis and invasion	metastasis	*Immunity*
anti-mitotic	methylation	*International journal of radiation oncology, biology, physics*
ATRX	methyltransferase	*JAMA*
bevacizumab	MGMT	*Journal of clinical neuroscience*
biomarker	microenvironment	*Journal of clinical oncology*
BRAF	midline	*Journal of neuro-oncology*
castleman	MRI	*Journal of neurosurgery*
cell biology	neuro-imaging	*Lancet*
cell signal	neurotoxicity	*Lancet oncology*
chemotherapy	next generation sequencing	*Medicine*
cholangiocarcinoma	oligodendroglioma	*Molecular neurobiology*
complications	p53	*Nature*
craniopharyngioma	palliative care	*Nature genetics*
diffuse astrocytoma	PD-1	*Nature medicine*
diffuse midline glioma	pediatric	*Nature methods*
DIPG	pilocytic astrocytoma	*Nature reviews cancer*
drug resistance	PNET	*Neuro-oncology*
EGFR	progression	*Neurosurgery*
ependymoma	quality of life	*Oncology letters*
epidemiology	radio	*Oncotarget*
epigenetics	recurrent	*PloS one*
epithelioid	schwannoma	*Science*
genetics	single cell	*Scientific reports*
germ cell tumor	spinal	*The New England journal of medicine*
glioblastoma	STAT	*World neurosurgery*
glioneuronal	stem cell	30 journals of interest
H3K9	targeted therapy	Abbreviations: 2-HG = 2-hydroxyglutarate. ATRX = alpha-thalassemia/mental retardation syndrome, nondeletion type, x-linked. BRAF = B-Raf. DIPG = diffuse intrinsic pontine glioma. EGFR = epidermal growth factor receptor. IDH = isocitrate dehydrogenase. MGMT = O6-methylguanine DNA methyltransferase. MRI = magnetic resonance imaging. PD-1 = programmed death-1. PNET = primitive neuroectodermal tumor. STAT = signal transducer and activator of transcription. TET2 = ten-eleven translocation 2. TT = tumor treating. WHO = world health organization.
hemangioblastoma	temozolomide	
histone	TET2	
IDH	thalamic	
immunology	translational	
inhibitor	TT	
K27M	tumor models	
leptomeningeal	WHO	
low-grade glioma		
79 fields of interest		

**Table 2:** Accuracy of the predictions by multiple regression analyses.

Regression Analyses	Accuracy A	Accuracy B	Accuracy C	Accuracy D
Linear prediction	70.6%	25.0% ^*^	38.3% ^**^	37.5% ^*^
Quadratic polynomial	54.4%	15.0% ^*^	30.0% ^*^	45.0% ^****^
Cubic polynomial	34.8%	28.8% ^*^	36.7% ^***^	20.0% ^*^

Accuracy A: The accuracy of prediction of the Δ-AII of the following year of data collection range within an error of 1.0-fold. B: The accuracy of prediction of the top 20 high Δ-AII fields of the following year of data collection range. C: The accuracy of prediction of the top 20 high Δ-AII fields of 2 years after data collection range. D: The accuracy of prediction of the top 20 high Δ-AII fields of 3 years after data collection range. Binominal test: * *p* > 0.05, ** *p* = 0.018, *** *p* = 0.034, **** *p* = 0.005.

**Table 3:** Top 20 hottest fields of neuro-oncology in 2019.

Predicted Rank	Fields
1	anti-mitotic
2	anaplastic oligodendroglioma
3	oligodendroglioma
4	TT
5	STAT
6	neurotoxicity
7	angiogenesis and invasion
8	radio
9	translational
10	cell biology
11	quality of life
12	palliative care
13	immunology
14	low-grade glioma
15	microenvironment
16	epigenetics
17	WHO
18	lymphoma
19	EGFR
20	biomarker
